# Prevalence of joint hypermobility in children with inguinal hernia

**DOI:** 10.1186/1546-0096-12-S1-P296

**Published:** 2014-09-17

**Authors:** Reza Shiari, Shima Salehi, Mehrnoush Hassas Yeganeh, Vadood Javadi Parvaneh

**Affiliations:** 1 Pediatric Rheumatology, Shahid Beheshti University Of Medical Sciences, Tehran, Iran, Islamic Republic Of

## Introduction

The Joint Hypermobility Syndrome (JHS) is a multi-system inherited connective tissue disorder caused by defective fibrous tissue matrix proteins such as collagen. Joint hypermobility has been occurred more frequently in children, with diminishing occurrence as age increases in population studies. The prevalence of hypermobility in children has been reported to be between 2.3 and 30%. Hypermobility is associated with weakness of abdominal wall and pelvic floor and predisposes to increased risk of hernia, hiatus hernia, and rectal prolaps.

## Objectives

The aim of this study is to determine the relation between of joint hypermobility syndrome (JHS) and unilateral and/or bilateral inguinal hernia in children.

## Methods

A Case-control study has been conducted in which 67 patients with inguinal hernia (unilateral and/or bilateral) accompanied by 86 healthy control group (age between 4 to 16 years old). The joint hypermobility was assessed by searching in all of the possible joints according to Beighton scoring system. Student’s *t*-test and Chi-squared test were used for the test of significance where applicable and *P* <0.05 was considered significant.

## Results

The prevalence of joint hypermobility in children with inguinal hernia (unilateral and/or bilateral) was 80.6% (54 of 67). The 60 out of 67 patients (89%) had unilateral and 1 (10.4%) cases had bilateral hernia.The prevalence of joint hypermobility in the group with unilateral hernia was 80%(48 of 60) and in bilateral hernia was 14.3% (1 of7). Comparison of the prevalence of disease between case and control groups indicated statistically significant difference (*P*<0.001).

## Conclusion

In our study, there was a significant correlation between joint hypermobility and both unilateral and/or bilateral inguinal hernia.

## Disclosure of interest

None declared

